# Heart rate variability: the comparison between high tension and normal tension glaucoma

**DOI:** 10.1007/s13167-017-0124-4

**Published:** 2018-02-22

**Authors:** Natalia Ivanovna Kurysheva, Tamara Yakovlevna Ryabova, Vitaliy Nikiforovich Shlapak

**Affiliations:** 1 Consultative-Diagnostic Department of Ophthalmological Center, Federal Medical and Biological Agency of the Russian Federation, Moscow, Russian Federation; 2 A.I. Burnazyan Federal Medical and Biophysical Center, Federal Medical and Biological Agency of the Russian Federation, Moscow, Russian Federation; 3 Ophthalmological Department of the Institute of Improvement of Professional Skills, Federal Medical and Biological Agency of the Russian Federation, Moscow, Russian Federation; 4 Science Center of Radiation and Chemical Safety and Hygiene, Medical and Biological Agency, Moscow, Russian Federation

**Keywords:** High tension glaucoma, Normal tension glaucoma, Ocular blood flow, Heart rate variability, Ocular perfusion pressure

## Abstract

**Relevance:**

Vascular factors may be involved in the development of both high tension glaucoma (HTG) and normal tension (NTG) glaucoma; however, they may be not exactly the same. Autonomic dysfunction characterized by heart rate variability (HRV) is one of the possible reasons of decrease in mean ocular perfusion pressure (MOPP).

**Purpose:**

To compare the shift of the HRV parameters in NTG and HTG patients after a cold provocation test (CPT).

**Methods:**

MOPP, 24-hour blood pressure and HRV were studied in 30 NTG, 30 HTG patients, and 28 healthy subjects. The cardiovascular fitness assessment was made before and after the CPT. The direction and magnitude of the average group shifts of the HRV parameters after CPT were assessed using the method of comparing regression lines in order to reveal the difference between the groups.

**Results:**

MOPP and minimum daily diastolic blood pressure were decreased in HTG and NTG patients compared to healthy subjects. There was no difference in MOPP between HTG and NTG before the CPT. However, all HRV parameters reflected the predominance of sympathetic innervation in glaucoma patients compared to healthy subjects (*P* < 0.05).

Before the CPT, the standard deviation of NN intervals (SDNN) of HRV was lower in HTG compared to NTG, 27.2 ± 4.1 ms and 35.33 ± 2.43 ms (*P* = 0.02), respectively. After the CPT, SDNN decreased in NTG by 1.7 ms and increased in HTG and healthy subjects by 5.0 ms and 7.09 ms, respectively (*P* < 0.05). The analysis of relative shift of other HRV parameters after the CPT also revealed a significant difference between NTG and HTG in regard to the predominance of sympathetic innervation in NTG compared to HTG.

**Conclusion:**

Patients with NTG have more pronounced disturbance of autonomic nervous system than HTG patients, which is manifested with the activation of sympathetic nervous system in response to CPT. This finding refers to the NTG pathogenesis and suggests the use of HRV assessment in glaucoma diagnosis and monitoring.

## Introduction

Vascular factors have been recognized to play an important role in glaucoma pathogenesis. According to the recent review, subjects with cardiovascular disease were 2.33 times more likely to develop rapidly progressive glaucoma disease despite significantly lower mean and baseline intraocular pressures (IOP) [1]. Moreover, the concept that vascular changes in the eye may be an early indicator of heart diseases is also discussed in literature [2].

It is believed that there are two groups of factors responsible for the development of glaucomatous optic neuropathy (GON) in case of normal IOP: (1) vascular dysfunction (dysregulation) leading to instable ocular blood flow of optic disc [3] and (2) mechanical dysfunction leading to damage of scleral membrane and infringement of axons in the optic nerve. It is generally recognized that high tension glaucoma (HTG) is most commonly characterized by mechanical dysfunction, while vascular dysregulation appears to be at the forefront in case of the normal tension glaucoma (NTG) development [4]. Meanwhile, it is known that instable ocular blood flow plays an important role in the development of both forms of glaucoma, including with high IOP [5–7]. The question that naturally arises: is insufficient blood supply to retina and optic nerve is more pronounced in NTG? In other words, are there more hemodynamic-related properties in NTG and are there any features of the general regulation of blood flow, which are typical for patients with NTG?

Recent studies have shown the role of vascular disturbances and vascular dysregulation in glaucoma progression, including NTG [8–13]. This unites glaucoma with such forms of pathology as migraine, vasospasm, arterial hypertension, and hypotension [9, 14].

Excessive activity of the sympathetic link of the autonomic nervous system (ANS) is one of the possible causes leading both to disturbance of blood supply to the ONH and to a decrease in ocular perfusion pressure (OPP) in the vessels of the optic nerve and choroid. Moreover, it has been shown in literature that the excessive activity of the sympathetic link of ANS is responsible for glaucoma progression due to an instable ocular blood flow [10].

It is believe that patients with an instable ocular blood flow respond stronger to psychological stress as it has been described in patients with primary vascular dysregulation (PVD) [4]. It has also been emphasized that any psychological stress leads to vascular dysfunction [15]. Cold stimulation is a well-established provocation test used for detecting abnormal vascular reactivity in patients with autonomic failures [16].

A significant reduction of retrobulbar blood flow in NTG has been described in literature [17, 18]. In addition, Kaiser et al. demonstrated that ocular blood flow was reduced in both NTG patients and in those HTG patients which progressed despite a normal IOP [17].

It has been recently revealed that the cold provocation test (CPT) may increase the ET-1 level in plasma in patients with NTG that reflected their vascular dysregulation [1]. This phenomenon may also indicate the imbalance of ANS that is manifested mostly during provocation tests, including CPT [19].

Patients with systemic autonomic dysfunction might be at higher risk for glaucoma progression due to higher susceptibility of the optic nerve to fluctuations of IOP or OPP. Heart rate variability (HRV) is a well-known tool that allows studying the autonomic modulation of the heart sympathovagal balance [20].

According to the literature, vascular risk factors are not exactly the same between HTG and NTG [2, 9, 21]. It was hypothesized that the vascular dysfunction would be more pronounced in NTG patients compared to HTG [16, 21, 22]. On the other hand, some authors reported that patients with HTG and NTG exhibit similar alterations in ocular and systemic circulation at the early stages of the disease [23].

There is no literature data concerning the influence of CPT on HRV in NTG compared to HTG patients.

The objective of this study is to compare the shift of HRV parameters in NTG and HTG patients after CPT.

## Materials and methods

### Study subjects

Eighty-eight eyes of 88 subjects (30 patients with early and moderate NTG, 30 patients with HTG, and 28 age-matched healthy subjects) were included in this study.

All patients were Caucasian.

HTG was diagnosed on the basis of characteristic changes in the optic disc detected by ophthalmoscopy, which was performed by one glaucoma specialist (NK) and confirmed by two other glaucoma specialists, pathological deviation from the normal neuroretinal rim, glaucomatous optic disc cupping, peripapillary atrophy, wedge-shaped defects of the retinal nerve fiber layer (RNFL) adjacent to the edge of optic disc, hemorrhages at the optic disc boundary, glaucomatous visual field (VF) loss on at least two consecutive tests, an open angle on gonioscopy (not less than 30°), and ametropia ≤ 0.5 diopter. IOP was higher than 21 mmHg.

The patients with the same criteria but with IOP of 21 mmHg or lower (without topical treatment), confirmed in repeated measurements on different days, were referred to the NTG group. All patients were followed up at our clinic for at least 4 years with visits at 3- to 5-month intervals and had no ocular pathology other than glaucoma.

The healthy participants were recruited from the people accompanying the patients and had IOP of less than 21 mmHg for both eyes, a normal Humphrey Swedish Interactive Threshold Algorithm 24-2 standard visual field with mean deviation (MD), and pattern standard deviation (PSD) within 95% limits of the normal reference. They also had a glaucoma hemifield test within 97% limits, a central corneal thickness ≥ 500 μm, a normal-appearing optic nerve head (ONH), a normal RNFL, an open anterior chamber angle as observed by gonioscopy, and no history of chronic ocular or systemic corticosteroid use. The age and race distribution of the healthy subjects matched that of the glaucoma patients.

Exclusion criteria were the following: large refractive errors (outside of ± 6.00 dpt sphere or 2.00 dpt cylinder), pupil diameter < 3 mm, systemic administration of beta-blockers and calcium-channel blockers, concomitant ocular disease (except for early cataract), chronic autoimmune diseases, diabetes mellitus, acute circulatory disorders in past medical history, and any concomitant diseases involving the administration of steroid drugs and antihypertensive medications. Patients with any significant cardiovascular, pulmonary, and metabolic conditions other than controlled systemic hypertension (BP < 140/90 mmHg) were excluded. A history of ocular arterial or venous obstruction (branch or central occlusion) and systemic conditions associated with venous congestion (e.g., heart failure) were also considered as exclusion criteria. The patients were instructed to avoid caffeine intake, smoking, and exercise for 5 h prior to the study visit.

If both eyes of a patient were eligible, one eye was randomly chosen. Those patients, who previously used antiglaucoma drops, were asked to discontinue the drug for a period of 21 days (drug washout period), while others were newly diagnosed glaucoma cases. The medical histories of all patients were carefully obtained with special attention paid to the signs of PVD (migraine, vasospasm, and neurocirculatory dystonia) and special questions were asked to reveal the symptoms of Flammer syndrome [21].

### Study examinations

All participants underwent complete ophthalmologic examinations including the best corrected acuity, slit lamp examination, IOP measurement using analyzer of biomechanical properties of eyes (Ocular Response Analyzer, ORA, Reichert Ophthalmic Instruments Inc., Depew, NY), gonioscopy, anterior chamber angle measurement (Visante OCT, Carl Zeiss, Germany), pachymetry (SP-100, Tomey, GmbH, Germany), dilated fundus biomicroscopy using 78-diopter lens, stereoscopic optic disc photography, and standard automated perimetry (SAP) using a Humphrey Field Analyzer (HFA, Carl Zeiss Meditec Inc., Dublin, CA) with SITA. Only reliable SAP results, which were defined as false-negative and false-positive responses of < 33% and fixation loss of < 20%, were eligible for the study. Glaucomatous VF defects were determined as having a cluster of three or more non-edge points with *P* < 0.05 and at least 1 point with *P* < 0.01 in the pattern deviation probability plot, PSD of less than 5%; or glaucoma hemifield test results outside normal limits. Both glaucoma and normal participants underwent SAP at least twice before this study.

The study included 24-h blood pressure (BP) monitoring: the automated measurement of BP for 24 h at fixed intervals according to a preset program. Measurements were made on an outpatient basis in the conditions of normal patient activity. The device measured the heart rate, systolic, and diastolic BP at set intervals using the oscillometric method, i.e., by analyzing pulse phenomena in a blood pressure cuff.

Mean ocular perfusion pressure (MOPP) was calculated on the basis of IOP and arterial BP measurements immediately before the optical coherence tomography (OCT) scanning and investigation of retrobulbar blood flow, after a 10-min resting period in a sitting position. Systemic BP was measured using the Riva Rocci technique. MOPP was calculated using the formula: MOPP = (2/3 diastolic BP + 1/3 systolic BP) × 2/ 3 − IOP.

BP was measured at rest in the sitting position. When measuring BP in the sitting position, the back had a support and the middle shoulder point was at the heart level (the fourth intercostal space). The measurement was made using Adyutor mechanical tonometer.

### OCT image acquisition and processing

All subjects also underwent optic disc area measurement at RTVue XR Avanti SD-OCT (Optovue, Inc., Fremont, CA, USA) using the traditional ONH scan. All the examinations for a particular subject were performed on the same day. OCT was performed in the macular area as well. The tracking mode was used.

The ganglion cell complex (GCC) thickness was determined with the GCC scanning protocol. The characteristics of GCC (global loss volume, GLV; focal loss volume, FLV) were also measured.

### HRV assessment

The cardiovascular fitness assessment was made to all patients before and after CPT using Rhythm-MET hardware-software complex developed by the Federal State Unitary Enterprise “Science Center of Radiation and Chemical Safety and Hygiene of the Medical and Biological Agency of the Russian Federation”.

Rhythm-MET hardware-software complex was used in the present study. The Rhythm-MET software and hardware system (registration certificate of the Federal Supervisory Agency for Healthcare and Social Development No. FSP 2009/04339 dated February 17, 2009) was developed to assess the state of health, to identify early arterial hypertension, and to assess functional state of the central nervous system, psychological, and emotional stability. The method of its work is based on a comprehensive analysis of HRV, systemic hemodynamics and autonomic regulation. Photoplethysmograms recorded from a phalanx with an infrared sensor, located in the microprocessor module of data input and processing, were used as the source of data on HRV and peripheral blood flow [24]. Cardiointervals obtained from photoplethysmograms were processed in accordance with the recommendations [25] for assessment of HRV parameters and their subsequent generalization, including hemodynamics parameters, as well as for assessment of functional status (FS) and functional reserves (FR) of the cardiovascular system according to the results of the examination at rest and after conducting CPT in order to form groups that are homogeneous in FS and FB. CPT also analyzed the parameters of time dependence of the amplitude of photoplethysmogram and recovery time after the test.

A generally accepted CPT was used as a provocation test in accordance to Weise et al. [26] with our modification. The testing procedure was the following: a patient’s hand was dipped into cold water (+ 4 °C) with small pieces of ice; moreover, the hand was covered with plastic ice bags for 30 s. The registration of RR intervals was made at the end of the CPT.

The following characteristics were taken into account in accordance with the international standard [25]:

- Standard deviation of NN-interval (SDNN): the parameter of HRV characterizing the total effect of autonomic blood circulation regulation. A reduction in SDNN reflects low HRV indicating a high tone of heart sympathetic activity. The decrease in SDNN reflects a decrease in HRV, which indicates an increase in the tone of heart sympathetic activity;
- The parameter of parasympathetic autonomic regulation activity (RMSSD);
- Total spectral power (TP): the parameter of the absolute activity level of regulatory systems;
- Power in the high frequency range (HF): the parameter of the spectral power of heart rate respiratory undulations reflecting the activity level of respiratory center. The high frequency band reflects fast changes in beat-to-beat variability due to parasympathetic activity;
- Power in the low frequency range (LF): the low frequency band is considered to be a fair approximation of sympathetic activity. The very low frequency band reflects mostly sympathetic stimulation. It also reflects the activity of the baroreflex mechanism ensuring the BP regulation;
- Autonomic regulation index (ARI): the parameter used to evaluate the activity of ANS. The increased ARI shows the activation of sympathetic regulation, but the decreased ARI shows the activation of parasympathetic regulation.
- The parameter characterizing the degree of tension of regulatory systems, in particular, is the activity level of the sympathetic regulation mechanisms (S). It may indicate the inotropic effect of enhancing of sympathetic nerve influences. This parameter reflects the activation of ergotropic regulation mechanisms and increases the intensity of energy processes.

### Statistical analysis

The used methods of statistical processing and analysis of obtained HRV results were aimed at establishing statistically significant differences between the NTG, HTG, and control groups both for the rest period and after CPT. These groups were formed on the basis of results of clinical examination, so they played the role of learning samples.

In this study, the method of comparing regression lines was applied [27]. The method of comparing regression lines has some advantages, for example, over the widely used method of comparing the mean group values due to the fact that it operates immediately with two indicators. In addition, it is also suitable for small samples. Based on the known physiological concepts, selection of parameters of autonomic regulation was made, for which a reliable separation of groups of patients from NTG and primary open-angle glaucoma (POAG) is possible. At the same time, the first necessary step in the search for these parameters was the assessment of the direction of the average group shifts, or the differences in the parameters after the CPT compared to the initial data. Next, the magnitude of the shift was taken into account (based on how much the index changed after the CPT)**.**

Finally, the selected parameters were statistically processed in order to reveal differences between the groups.

The conclusion that the response to cold provocation test in patients with NTG and POAG is statistically reliably different was done on the basis of the negative result of the statistical test for the coincidence of the regression lines.

The procedure of comparing regression lines includes the comparison of coefficients of slope *b* (slope ratio), coefficients of shear *a*, and the comparison of lines in general [28].

Among all possible regression lines, we used that one, which corresponds to the well-known theory of initial value in physiology [29]. According to this theory, direction and value of endogenous and exogenous effect depend on the initial metabolism and the state of regulatory body systems before CPT. HRV values reflect the peculiarities of the state of regulatory mechanisms of cardiovascular system in NTG, HTG patients, and control subjects.

Taking into account the abovementioned theory, regression lines are as follows:

ΔHRV_NTG_ = *a*_NTG_ + D *b*_NTG_∙HRV_NTG,rest_, ΔHRV_HTG_ = *a*_HTG_ + *b*_HTG_∙HRV_HTG,rest_, where ΔHRV_NTG,CPT_ and ΔHRV_HTG,CPT_ are shifts of the studied HRV parameter after CPT for the NTG and HTG groups, respectively; *a*_NTG_, *b*_NTG_ and *a*_HTG_, *b*_HTG_ are the coefficients of regression lines for NTG and HTG groups, respectively, whereby *a* and *b* are the coefficients of regression characterizing the degree of conjugation and the proportionality of shift in characteristics. The coefficient *b* reflects the strength of connection, i.e., the slope of the line and the intersection of the ordinate axes, the coefficient *a* determines the position of the line in the coordinate axes.

HRV_NTG,rest_ and HRV_HTG,rest_ are HRV parameters relating to rest for the NTG and HTG groups, respectively; the shift in HRV parameters is determined by the following ratios: ΔHRV_NTG,CPT_ = HRV_NTG,CPT_ − HRV_NTG,rest_, ΔHRV_HTG,CPT_ = HRV_HTG,CPT_ − HRV_HTG,rest_.

The conclusion on statistically significant difference of regression lines, and, therefore, on the difference of the compared groups is built on the basis of comparing the calculated statistics with the values of Fisher’s test (for coefficient *b*) and the values of Student’s test (for coefficient a and comparison of lines in general).

In this study, the regression lines for the NTG, HTG, and control groups were compared using the statistical software package considering the specific character of medical and biological tasks [28].

The following designations were applied to the checks of statistical significance of difference of regression lines: “1”—to the criterion of coincidence of regression lines in general, “2”—to the criterion of comparison of line slopes (*b*), and “3”—to the criterion of comparison of shifts (*a*).

Standard methods of descriptive statistics were used as follows: *T* tests, methods of non-parametric statistics—Wilcoxon test, Mann-Whitney *U* test, implemented in the corresponding statistics packages (IBM SPSS Statistics v 21, StatPlus).

Parameters with *Р* < 0.05 were considered statistically significant. Since a number of parameters (GCC, GLV, systolic, and mean perfusion pressure) depended on the anterior-posterior axis and the age of the subjects, we carried out an adjustment for these parameters on the basis of the linear regression model.

## Results

The patient characteristics are given in Table 1.

**Table 1:** Characteristics of the studied groups

Parameter	Healthy eyes	*P*	NTG eyes	P**	HTG eyes	P***
Age, years	63.8 (9.8)	0.621	63.4 (5.8)	0.26	62.5 (4.3)	0.745
Systolic BP, mm Hg	127.4 (13.4)	0.043	138.2(7.3)	0.055	131.0(5.2)	0.056
Diastolic BP, mm Hg	81.0 (9.3)	0.04	75.5 (8.3)	0.84	74.9 (5.8)	0.03
Minimum daily diastolic BP, mm Hg	38.0 (7.1)	0.02	30.0 (6.2)	0.09	32.0 (8.6)	0.05
Corneal compensated IOP, mm Hg	14.8 (3.6)	0.22	14.1 (3.4)	0.01	24.5 (5.2)	0.02
MOPP, mm Hg	61.1 (8.5)	0.023	53.1 (8.1)	0.051	51.3 (7.3)	0.021
MD, dB	− 0.7 (2.12)	0.001	− 5.8 (4.2)	0.31	− 6.5 (3.6)	< 0.001
PSD, dB	1.20 (0.58)	0.005	3.79 (1.45)	0.25	4.09 (1.12)	0.05
RNFL, μm	103.9 (7.1)	0.002	85.3 (5.1)	0.34	83.6 (5.1)	0.001
Savg, μm	119.1 (9.9)	0.003	90.9 (6.2)	0.24	92.3 (4.8)	0.03
Iavg, μm	125.1 (10,2)	0.003	97.8 (6.0)	0.09	93.7 (4.0)	0.03
GCC, μm	99.3 (8.2)	0.005	80.3 (6.2)	0.21	76.2 (5.52)	0.004
FLV, %	0.18 (0.09)	0.003	3.57 (1.12)	0.08	5.02 (2.14)	0.002
GLV, %	1.61 (1.86)	0.002	8.35 (1.86)	0.34	7.33 (2.36)	0.001
Axial length, mm	23.1 (1.3)	0.675	23,3 (1.4)	0.45	23,6 (1.8)	0.33
Corneal thickness, μm	532.6 (20.2)	0.923	536 (21.3)	0.38	530 (18.1)	0.423

The table shows the mean values and standard deviation (in parentheses), the exact two-sided Mann-Whitney *U* test (P*) between the healthy eyes (control group) and the NTG patients, P** between the NTG and HTG groups, and total P*** obtained when comparing these three groups by means of Kruskal-Wallis test by ranks. All abbreviations are given in the list of abbreviations.

No significant differences in SBP, DBP, minimum DBP, and MOPP were identified between NTG and HTG patients across the 24-h measurement period though there were a significant difference between both the glaucoma groups and healthy subjects (Table 1).

The parameters of HRV in all studied groups are represented in Table 2. Its left side shows the mean group values of HRV parameters in the NTG, HTG, and control groups. Moreover, the table contains the data on statistical significance (*P*) of intragroup differences in parameters after CPT (columns 4, 7, and 10) for each group. The right side of Table 2 gives the information on the results of estimates of statistical significance of intragroup differences of NTG and HTG at rest (column 11) and after CPT (column 12).

**Table 2:** The mean group values of HRV before and after cold provocation test in the normal tension glaucoma (NTG), high tension glaucoma (HTG), and control groups

HRV parameters	NTG group			HTG group			Control group			Statistical significance of differences between groups (*Р*)					
										NTG_rest_ ⇕ HTG_rest_	NTG_CPT_ ⇕ HTG_CPT_	NTG_rest_ ⇕ Control_rest_	NTG_CPT_ ⇕ Control_CPT_	HTG_rest_ ⇕ Control_rest_	HTG_CPT_ ⇕ Control_CPT_
	Rest	CPT	*P*	Rest	CPT	*P*	Rest	CPT	*P*	*P*	*P*	*P*	*P*	*P*	*P*
1	2	3	4	5	6	7	8	9	10	11	12	13	14	15	16
SDNN (ms)	35.3 ± 12.4	33.6 ± 11.3	0.08	27.2 ± 10.0	32.2 ± 13.2	*0.014*	42.03 ± 5.12	49.12 ± 9.02	*0.004*	*0.016*	0.43	0.20	*0.004*	*0.001*	*0.001*
RMSSD (ms)	34.2 ± 11.3	35.5 ± 16.4	0.71	28.1 ± 12.2	32.3 ± 16.6	0.29	35.6 ± 5.1	35.7 ± 16.2	0.98	*0.046*	0.39	0.17	0.23	*0.014*	*0.038*
HF (ms^2^)	242.4 ± 169.3	250.2 ± 10.1	0.78	216.4 ± 87.2	253.33 ± 10.12	0.78	307.12 ± 304	400.1 ± 149.3	0.085	*0.045*	0.37	0.58	*0.003*	0.35	*0.024*
LF (ms^2^)	233.2 ± 132.1	272.4 ± 180.2	0.82	236.1 ± 117	310.1 ± 191.3	*0.024*	743.5 ± 292.1	716.6 ± 360.3	0.90	*0.03*	0.81	0.96	0.54	0.23	0.18
S	1.61 ± 0.28	1.75 ± 0.37	0.95	2.40 ± 0.61	2.21 ± 0.77	0.65	1.04 ± 0.43	1.29 ± 0.42	*0.02*	0.14	0.80	0.09	*0.001*	*0.001*	*0.001*
ARI	315.3 ± 86.12	357.42 ± 129.1	0.91	453.2 ± 111	405.7 ± 104.	0.26	169.1 ± 35.2	189.38 ± 59.63	0.09	0.16	0.62	0.14	*0.001*	*0.001*	*0.001*
TP	1082 ± 924	964 ± 571	0.65	655 ± 528	918 ± 688	0.14	1773 ± 1542	2339 ± 2063	0.27	0.018	0.29	0.10	*0.009*	*0.001*	*0.001*

Mann-Whitney *U* test was used to assess the statistical significance of the groups. Italic value demonstrate significant difference of the compared values (for visual clarity)

*CPT*, cold provocation test; *NTG*_*rest*_, *HTG*_*rest*_, collective designation of belonging of values of NTG and HTG groups at rest, respectively; *NTG*_*CPT*_, *HTG*_*CPT*_, collective designation of belonging of values of NTG and HTG groups after CPT, respectively; ⇕, symbol denoting the comparison of HRV values of two groups

The statistical significance of intragroup differences of the HTG, NTG, and control groups at rest and after CPT is given in columns 13 and 14. The columns 15 and 16 contain the result of estimates of statistical significance of intragroup differences of the HTG and control groups.

The NTG and HTG groups are significantly different from the control group according to the mean group values of HRV parameters. For example, according to CPT results (column 14, Table 2), the NTG group differs from the HTG and control groups both at rest and after CPT (columns 15 and 16). At the same time, there are significant differences between the NTG and HTG groups only at rest (column 11). This was one of the reasons to use other methods of statistical processing, in particular, the method of comparing regression lines, to analyze the intragroup differences.

**Fig. 1:**
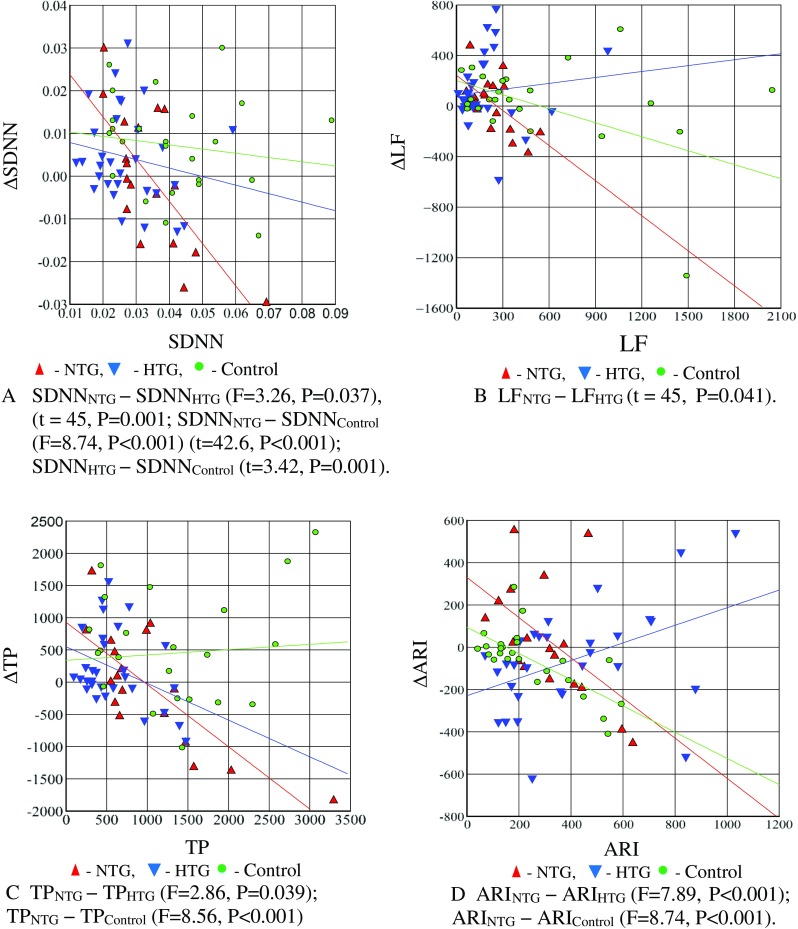
Comparison of regression lines for HRV parameters in the NTG, HTG, and control subject groups. Note: the slope of regression lines characterizes the relation between two variables: shift in HRV parameter (ΔHRV = HRV_CPT_ − HRV_rest_) after CPT and its value at rest (HRV_rest_). A positive slope of the line indicates an increase in ΔHRV at HRV_rest_ increase; a negative slope reflects a decrease in ΔHRV at HRV_rest_ increase.

To compare the NTG and HTG groups, Fig. 1 shows the regression lines for those HRV parameters, whose regression lines are significantly different and the results of the intergroup differences in HRV indicators, revealed by the method of comparison of regression lines, are represented in Table 3.

**Table 3:** The intergroup differences in HRV indicators, revealed by the method of comparison of regression lines

No.	Compared indicators	Values of the difference criteria	Significance level
1	SDNN_NTG_ and SDNN_HTG_	*F*_Fisher_ = 3.26	*P* = 0.037
		*t*_Student_ = 45	*P* = 0.001
2	SDNN_NTG_ and SDNN_control_	*F*_Fisher_ = 8.74	*P* < 0.001
		*t*_Student_ = 42.6	*P* < 0.001
3	SDNN_HTG_ and SDNN_control_	*t*_Student_ = 3.42	*P* = 0.001
4	LF_NTG_ and LF_HTG_	*F*_Fisher_ = 3.21	*P* = 0.05
5	TP_HTG_ and TP_HTG_	*F*_Fisher_ = 2.86	*P* = 0.039
6	TP_HTG_ and TP_control_	*F*_Fisher_ = 8.56	*P* < 0.001
7	ARI_NTG_ and ARI_HTG_	*F*_Fisher_ = 7.89	*P* < 0.001
8	ARI_NTG_ and ARI_control_	*F*_Fisher_ = 8.74	*P* < 0.001

The obtained results relating to HRV parameters confirm the earlier assumption on the difference in groups:

NTG and HTG in each of the pairs of parameters of these groups: (SDNN, ΔSDNN), (LF, ΔLF) (ARI, ΔARI), and (TP, ΔTP);

- NTG and Control in the pairs of parameters: (SDNN, ΔSDNN), (ARI, ΔARI).
- HTG and Control in the pairs of parameters: (SDNN, ΔSDNN), (ARI, ΔARI).

## Discussion

To the best of our knowledge, this is the first study that has revealed the difference in HRV parameters in NTG and HTG patients both at rest and in response to CPT. Our data confirmed the predominance of the sympathetic ANS at rest in NTG compared to HTG and the shift towards sympathetic activity after CPT, which was more pronounced in NTG compared to HTG and control subjects.

It has been recognized that POAG according to its strict definition includes HTG and NTG. As far as HTG and NTG are concerned, they appear to be a continuum of glaucomatous process, in which the underlying mechanisms shifts from predominantly elevated IOP in HTG to hemodynamic change in NTG. In other words, both HTG and NTG are related to hemodynamics, but it was hypothesized that the evidence of vascular dysfunction would be more pronounced in NTG patients. One of the possible reasons for this is autonomic dysfunction that may contribute to unstable or fluctuating blood pressure and thereby may induce the dysfunction of autoregulation leading to glaucoma development and progression [3].

The autonomic dysfunction in patients with NTG using short-term and a 24-h heart rate variability analysis have been reported by different authors [10, 30–33]. However, the literature data on autonomic dysfunction in HTG and NTG are controversial. According to Riccadonna M. et al., HRV and nocturnal diastolic BP variability were reduced in NTG compared to HTG [31]. Furthermore, these differences were more prominent in more severe clinical forms of NTG. The authors suggested a correlation between the extent of autonomic disorder and severity of glaucoma.

Brown et al. evaluated baroreflex control of the heart and blood vessels in HTG and NTG patients using the sinusoidal neck suction. They revealed that the response of ANS in the healthy subjects was significantly greater than that in glaucoma patients. However, they did not detect any difference between NTG and HTG. According to their data, the decreased sympathetic and parasympathetic modulation during baroreceptor stimulation in the patients with HTG and NTG suggested that autonomic dysfunction that may contribute to the pathogenesis of both diseases [32].

Mroczkowska et al. compared HTG and NTG patients with early stage of the disease using 24-h ambulatory blood pressure monitoring and measuring peripheral pulse-wave analysis, and carotid intima-media thickness. Retinal vascular reactivity to flicker light was assessed as well. Though the patients of both glaucoma groups exhibited similar alterations in ocular and systemic circulation compared to healthy subjects, no significant differences were found in retinal arterial or venous flicker response, nocturnal blood pressure, systemic arterial stiffness, and intima-media thickness between HTG and NTG patients [23].

According to Bossuyt et al., OPP was significantly reduced in HTG and NTG patients compared with controls, suggesting that perfusion-related vascular alterations are likely to be playing a part in the pathogenesis of both conditions [34].

These findings allow us to propose that it is unreasonable to differentiate NTG and HTG.

On the other hand, there are some important differences between HTG and NTG. For example, the VF progression pattern in NTG is reported to be different when compared with other types of glaucoma [35]. Notably, NTG eyes progressed more frequently in the central region of the VF, and this response was related to unstable or large fluctuations of 24-h mean ocular perfusion pressure, and excessive nocturnal dips of systemic blood pressure (BP) [10, 36]. Hence, a separation between NTG and HTG is still usual in clinical practice [37].

We have recently revealed that ocular blood flow was significantly reduced both in NTG and HTG compared to healthy subjects. The reduction of arterial ocular blood flow was more significant in HTG than in NTG while the lower venous blood flow was detected in NTG patients [18]. It was emphasized in literature that the reduction of blood flow velocities in the central retinal vein and central retinal artery was significantly associated with glaucoma progression both in NTG and HTG patients with well controlled IOP (21 mmHg or less) [17]. It means that circulatory disorders may occur both in NTG and HTG despite the IOP level. One of the reasons is the increased sympathetic neural activity (SNA). It leads to an increase in vascular resistance and especially under circumstances of the endothelial dysfunction may have circulatory implications relevant to glaucoma pathogenesis. SNA causes an increase of heart rate, stroke volume, and vasoconstriction. It regulates the circadian variation of BP and is closely linked to nocturnal dipping.

In the present study, we observe a significant dipping of diastolic BP both in NTG and HTG patients compared to healthy subjects. This may be a consequence of the activation of the sympathetic innervation. Chronic increased SNA can lead to arterial and cardiac remodeling, endothelial dysfunction, increased tissue oxygen demand, and subsequent decreasing of the ischaemia threshold in all organs, including the eye. There is the evidence of the presence of the choroidal neuroplexus represented by numerous internal autonomic ganglia, forming the autonomous perivascular network around the choroidal vessels [6]. It is believed that it plays vasodilatory function aimed at enhancing the ocular blood flow. Apparently, the vascular mechanisms of failure of optic nerve and retinal trophism and their autonomic regulation play a significant role in ocular physiology and pathophysiology in general and, particularly, in glaucoma. Vasoconstriction occurs in the setting of the predominance of sympathoadrenal influences on arterioles and capillaries, as well as due to the decreased activity of parasympathetic influences on retinal vessels.

Altered ocular blood flow or reduced visual field sensitivity during sympathetic provocation tests has been demonstrated in POAG patients [33, 38, 39].

The present study has identified the evidence of altered MOPP both in HTG and NTG patients compared to healthy subjects. However, there were no difference in the MOPP between HTG and NTG that is consistent with previous research [23, 31, 38]. Such findings suggest that a considerable overlap may exist in the development of HTG and NTG, especially at the early stage of the disease [23, 40]. From this point of view, it has been assumed that provocation tests may be needed to reveal alterations in cardiovascular function in NTG patients [34]. In the present study, we applied CPT to reveal the difference between NTG and HTG patients.

Before CPT, a significant difference was revealed for all HRV parameters at rest between both the glaucoma groups and between HTG and control subjects. The CPT confirmed a significant difference between glaucoma patients and control subjects.

This was the reason to compare regression lines for analyzing the intergroup differences. The mentioned method using the example of comparing the NTG and HNG groups by SDNN shows that significance of differences in groups can be established immediately by two characteristics: initial values ​of the parameter at rest and its changes as a result of the reaction to CPT.

As the result of this fact, we revealed the most important data of this study: a considerable increase in the activity of the sympathetic ANS in NTG patients in response to the CPT. The changes of the basic HRV parameters (SDNN, HF, LF, S, and ARI) after the CPT emphasize a significant difference between HTG and NTG patients. It is known that an increase of the sympathetic ANS in response to the provocation tests is typical for people with PVD. The development of NTG is associated with possible PVD [2, 21]. However, currently this fact is not absolutely certain, and therefore, NTG is considered to be a form of an open-angle glaucoma.

Although the role of PVD in the pathogenesis of GON has been discussed for many years, only recent studies due to the use of modern technologies could prove that patients with NTG, but not healthy individuals, suffer from the retinal blood flow autoregulation failure in the conditions of provocation tests [5]. From this point of view, the dysfunction of the autonomic blood flow regulation seems to be of high importance and its study attracts attention of the researches. Wierzbowska et al. studied HRV in NTG and revealed the sympathovagal balance of ANS in NTG patients that shifted towards sympathetic activity with no change of 24-h pattern of BP variability as compared to the healthy subjects [33]. Na et al. also observed significantly decreased SDNN values in patients with NTG [30].

Park and co-authors studied the NTG patients with different types of HRV and reported that VF progression in patients with sympathetic predominance is faster than that in patients with higher HRV. The authors concluded that the autonomic dysfunction, especially the decrease of SDNNs, was a predictor of central VF progression in NTG [10].

The new data, which testify to the influence of vascular factors on the development of NTG, are especially relevant. It can be assumed that violations of autonomic innervation underlying PVD are an important cause of NTG development, but not its specific feature. Being present in POAG patients, including HTG, an imbalance of the ANS can be also considered as a risk factor for unfavorable course of GON. In any case, the results obtained convincingly demonstrate the role of PVD in the NTG pathogenesis. Our results that demonstrate the predominance of SNA in NTG patients may be used for distinguishing NTG from HTG.

This conclusion has an important practical implication for detecting NTG (or if it is suspected), determining the prognosis and choosing more appropriate therapy, as well as making recommendations to patients concerning the proper lifestyle. Further studies are needed to verify our findings as well as studies on any therapies that favorably influence ANS activity in patients with glaucoma.

Our study has several limitations that must be acknowledged. First, we did not study the progression pattern of the disease and we did not analyze the relation between the shift of HRV parameters after CPT and functional loss in HTG and NTG, although it might reveal the influence of HRV on glaucoma progression and its difference between the two studied glaucoma groups.

Second, we did not evaluate the circulatory parameters and their relation to the HRV parameters.

Third, we did not assess test-retest variability, though it is known that HRV is a moderately to fairly good reliable measurement [41].

Fourth, MOPP was calculated using the formula: MOPP = (2/3 diastolic BP + 1/3 systolic BP) × 2/3 − IOP, though it has been emphasized in literature, that retinal venous pressure is very often markedly higher than IOP, that means that our calculation may not be adequate enough in some patients [42].

However, our study has an advantage over other studies on HRV in NTG and POAG as we did not include patients on antihypertensive medication or topical medications that could influence BP and heart rate values.

In summary, we found out that patients with NTG have more pronounced disturbance of the autonomic nervous system than HTG patients that is manifested by the activation of sympathetic nervous system in response to the CPT. This finding refers to the NTG pathogenesis and suggests the use of HRV assessment in glaucoma diagnosis and monitoring.

## Abbreviations

ANS: autonomic nervous system
ARI: autonomic regulation index
BP: blood pressure
CPT: cold provocation test
DBP: diastolic blood pressure
FLV: focal loss volume
FR: functional reserves
FS: functional status
GCC: ganglion cell complex
GLV: global loss volume
GON: glaucomatous optic neuropathy
HF: high frequency range
GON: glaucomatous optic neuropathy
HTG: high tension glaucoma
HRV: heart rate variability
ILM: internal limiting membrane
IOP: intraocular pressure
LF: low frequency range
MD: mean deviation
MOPP: mean ocular perfusion pressure
NTG: normal tension glaucoma
OCT: optical coherence tomography
ONH: optic nerve head
OPP: ocular perfusion pressure
POAG: primary open-angle glaucoma
PSD: pattern standard deviation
PVD: primary vascular dysregulation
RMSSD: root mean square of the successive differences
RNFL: retinal nerve fiber layer
RPE: retinal pigment epithelium
SAP: standard automated perimetry
SBP: systolic blood pressure
SDNN: standard deviation of the NN intervals
SD-OCT: spectral-domain optical coherence tomography
SNA: sympathetic neural activity
TP: total spectral power
VF: visual field
